# (*E*)-*N*′-(5-Chloro-2-hydroxy­benzyl­idene)-*p*-toluene­sulfonohydrazide

**DOI:** 10.1107/S1600536808038695

**Published:** 2008-11-22

**Authors:** Reza Kia, Hoong-Kun Fun, Hadi Kargar

**Affiliations:** a X-ray Crystallography Unit, School of Physics, Universiti Sains Malaysia, 11800 USM, Penang, Malaysia; b Department of Chemistry, School of Science, Payame Noor University (PNU), Ardakan, Yazd, Iran

## Abstract

The title compound, C_14_H_13_ClN_2_O_3_S, features an intra­molecular O—H⋯N hydrogen bond which generates an *S*(6) ring motif. Inter­molecular N—H⋯O hydrogen bonds and C—H⋯O close contacts link neighbouring mol­ecules forming *R*_2_^2^(13) ring motifs. In the crystal structure, mol­ecules are further linked by C—H⋯Cl inter­actions, forming one-dimensional extended chains along the *c* axis. The dihedral angle between the two benzene rings is 86.06 (3)°. The crystal structure is further stabilized by weak inter­molecular π–π inter­actions [inter­planar stacking distance = 3.357 (7) Å].

## Related literature

For related structures and applications, see, for example: Kayser *et al.* (2004 ▶); Tierney *et al.* (2006 ▶); Tabatabaee *et al.* (2007 ▶); Ali *et al.* (2007 ▶); Mehrabi *et al.* (2008 ▶); Kia *et al.* (2008 ▶). For the values of bond lengths, see: Allen *et al.* (1987 ▶). For hydrogen-bond motifs, see: Bernstein *et al.* (1995 ▶).
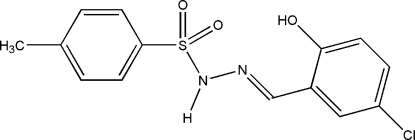


## Experimental

### 

#### Crystal data

C_14_H_13_ClN_2_O_3_S
*M* *_r_* = 324.78
Monoclinic, 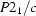

*a* = 15.7454 (3) Å
*b* = 9.8338 (2) Å
*c* = 9.8455 (2) Å
β = 105.941 (1)°
*V* = 1465.83 (5) Å^3^
*Z* = 4
Mo *K*α radiation
μ = 0.41 mm^−1^
*T* = 100.0 (1) K
0.45 × 0.38 × 0.31 mm

#### Data collection

Bruker SMART APEXII CCD area-detector diffractometer
Absorption correction: multi-scan (**SADABS**; Bruker, 2005 ▶) *T*_min_ = 0.836, *T*_max_ = 0.883
16498 measured reflections
5274 independent reflections
4761 reflections with *I* > 2σ(*I*)
*R*_int_ = 0.020

#### Refinement

*R*[*F* ^2^ > 2σ(*F* ^2^)] = 0.031
*wR*(*F* ^2^) = 0.093
*S* = 1.10
5274 reflections
199 parameters
H atoms treated by a mixture of independent and constrained refinement
Δρ_max_ = 0.44 e Å^−3^
Δρ_min_ = −0.38 e Å^−3^

### 

Data collection: *APEX2* (Bruker, 2005 ▶); cell refinement: *APEX2*; data reduction: *SAINT* (Bruker, 2005 ▶); program(s) used to solve structure: *SHELXTL* (Sheldrick, 2008 ▶); program(s) used to refine structure: *SHELXTL*; molecular graphics: *SHELXTL*; software used to prepare material for publication: *SHELXTL* and *PLATON* (Spek, 2003 ▶).

## Supplementary Material

Crystal structure: contains datablocks global, I. DOI: 10.1107/S1600536808038695/pk2133sup1.cif

Structure factors: contains datablocks I. DOI: 10.1107/S1600536808038695/pk2133Isup2.hkl

Additional supplementary materials:  crystallographic information; 3D view; checkCIF report

## Figures and Tables

**Table 1:** Hydrogen-bond geometry (Å, °)

*D*—H⋯*A*	*D*—H	H⋯*A*	*D*⋯*A*	*D*—H⋯*A*
O1—H1*O*1⋯N1	0.75 (2)	2.00 (2)	2.6690 (13)	149 (2)
N2—H1*N*2⋯O2^i^	0.881 (16)	1.961 (17)	2.8375 (12)	172.8 (17)
C7—H7*A*⋯O1^i^	0.93	2.59	3.3679 (14)	142
C10—H10*A*⋯Cl1^ii^	0.93	2.82	3.7256 (12)	164
C12—H12*A*⋯O3^iii^	0.93	2.48	3.3549 (14)	157

Symmetry codes: (i) 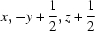; (ii) 
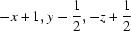
; (iii) 
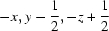
.

**Fig. 1.**
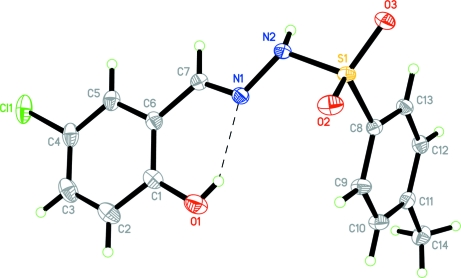
The molecular structure of the title compound, showing 50% probability displacement ellipsoids and the atomic numbering scheme. The intramolecular hydrogen bond is shown as a dashed line.

**Fig. 2.**
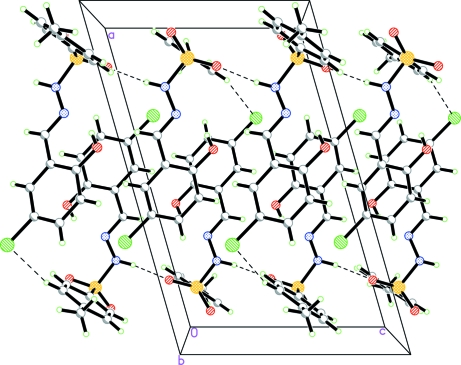
The crystal packing viewed down the b-axis, showing one-dimensional extended chains along the c-axis. Intermolecular interactions are shown as dashed lines.

## supplementary crystallographic information

### Comment

Sulfonamides were the first class of antimicrobial agents to be discovered. Sulfonamides (sulfanilamide, sulfamethoxazole, sulfafurazole) are structural analogues of *p*-aminobenzoic acid (PABA) and compete with PABA to block its conversion to dihydrofolic acid (Kayser *et al.*, 2004). These agents are generally used in combination with other drugs (usually sulfonamides) to prevent or treat a number of bacterial and parasitic infections (Tierney *et al.*, 2006). With regard to all of the above important features, we report the crystal structure of the title compound.

The title compund (Fig. I), is a novel sulfonamide derivative. Bond lengths (Allen *et al.*, 1987) and angles are within the normal ranges and are comparable with those in related structures (Kia *et al.*, 2008; Mehrabi *et al.*, 2008; Ali *et al.* 2007). An intramolecular O—H···N hydrogen bond generates *S*(6) ring motif, and the molecule adopts a 'vault' shape. Intermolecular N—H···O and C—H···O interactions link neighbouring molecules by *R*_2_^2^(13) ring motifs. The two benzene rings make a dihedral angle of 86.06 (3)°. In the crystal structure, molecules are linked together by intermolecular C—H···Cl, N—H···O and C—H···O interactions, forming one-dimensional extended chains along the *c* axis. The crystal structure is further stabilized by weak intermolecular π-π interactions [interplanar distance = 3.357 (7) Å].

### Experimental

The synthetic method has been described earlier (Kia *et al.*, 2008). Single crystals suitable for *X*-ray diffraction were obtained by evaporation of an ethanol solution at room temperature.

### Refinement

The H atoms bound to O1 and N2 were found in a difference Fourier map and refined freely. The rest of the hydrogen atoms were positioned geometrically and refined using a riding model. A rotating group model was used for the methyl hydrogens. The highest residual peak (0.44 e.Å^-3^) is located 0.66 Å from O2 and the deepest hole (-0.38 e.Å^-3^) is located 0.57 Å from S1.

### Crystal data

**Table:**

C_14_H_13_ClN_2_O_3_S	*F*_000_ = 672
*M**_r_* = 324.78	*D*_x_ = 1.472 Mg m^−^^3^
Monoclinic, *P*2_1_/*c*	Mo *K*α radiation λ = 0.71073 Å
Hall symbol: -P 2ybc	Cell parameters from 9969 reflections
*a* = 15.7454 (3) Å	θ = 2.7–36.2º
*b* = 9.8338 (2) Å	µ = 0.41 mm^−^^1^
*c* = 9.8455 (2) Å	*T* = 100.0 (1) K
β = 105.9410 (10)º	Block, colourless
*V* = 1465.83 (5) Å^3^	0.45 × 0.38 × 0.31 mm
*Z* = 4	

### Data collection

**Table:**

Bruker SMART APEXII CCD area-detector diffractometer	5274 independent reflections
Radiation source: fine-focus sealed tube	4761 reflections with *I* > 2σ(*I*)
Monochromator: graphite	*R*_int_ = 0.020
*T* = 100.0(1) K	θ_max_ = 32.5º
φ and ω scans	θ_min_ = 1.3º
Absorption correction: multi-scan(*SADABS*; Bruker, 2005)	*h* = −16→23
*T*_min_ = 0.836, *T*_max_ = 0.883	*k* = −11→14
16498 measured reflections	*l* = −14→13

### Refinement

**Table:**

Refinement on *F*^2^	Secondary atom site location: difference Fourier map
Least-squares matrix: full	Hydrogen site location: inferred from neighbouring sites
*R*[*F*^2^ > 2σ(*F*^2^)] = 0.031	H atoms treated by a mixture of independent and constrained refinement
*wR*(*F*^2^) = 0.093	*w* = 1/[σ^2^(*F*_o_^2^) + (0.0458*P*)^2^ + 0.5147*P*] where *P* = (*F*_o_^2^ + 2*F*_c_^2^)/3
*S* = 1.10	(Δ/σ)_max_ = 0.001
5274 reflections	Δρ_max_ = 0.44 e Å^−^^3^
199 parameters	Δρ_min_ = −0.38 e Å^−^^3^
Primary atom site location: structure-invariant direct methods	Extinction correction: none

### Special details

**Table:**

Experimental. The low-temperature data was collected with the Oxford Cryosystems Cobra low-temperature attachment.
Geometry. All e.s.d.'s (except the e.s.d. in the dihedral angle between two l.s. planes) are estimated using the full covariance matrix. The cell e.s.d.'s are taken into account individually in the estimation of e.s.d.'s in distances, angles and torsion angles; correlations between e.s.d.'s in cell parameters are only used when they are defined by crystal symmetry. An approximate (isotropic) treatment of cell e.s.d.'s is used for estimating e.s.d.'s involving l.s. planes.
Refinement. Refinement of *F*^2^ against ALL reflections. The weighted *R*-factor *wR* and goodness of fit *S* are based on *F*^2^, conventional *R*-factors *R* are based on *F*, with *F* set to zero for negative *F*^2^. The threshold expression of *F*^2^ > σ(*F*^2^) is used only for calculating *R*-factors(gt) *etc*. and is not relevant to the choice of reflections for refinement. *R*-factors based on *F*^2^ are statistically about twice as large as those based on *F*, and *R*- factors based on ALL data will be even larger.

### Fractional atomic coordinates and isotropic or equivalent isotropic displacement parameters (Å^2^)

**Table:**

	*x*	*y*	*z*	*U*_iso_*/*U*_eq_	
Cl1	0.694874 (18)	0.12795 (3)	0.64129 (3)	0.03146 (8)	
S1	0.146818 (15)	0.24140 (2)	0.10615 (2)	0.01365 (6)	
O1	0.41411 (6)	0.06452 (10)	0.11049 (9)	0.02515 (17)	
O2	0.17263 (6)	0.28771 (8)	−0.01531 (8)	0.02080 (15)	
O3	0.07539 (5)	0.30583 (8)	0.14463 (8)	0.01865 (14)	
N1	0.31124 (5)	0.20818 (9)	0.23227 (9)	0.01581 (15)	
N2	0.23293 (5)	0.26798 (9)	0.24403 (9)	0.01551 (15)	
C1	0.47774 (7)	0.08266 (11)	0.23401 (11)	0.02019 (19)	
C2	0.56194 (8)	0.03165 (13)	0.24406 (13)	0.0267 (2)	
H2A	0.5731	−0.0121	0.1670	0.032*	
C3	0.62907 (8)	0.04587 (13)	0.36843 (14)	0.0277 (2)	
H3A	0.6850	0.0113	0.3751	0.033*	
C4	0.61217 (7)	0.11203 (12)	0.48286 (13)	0.0234 (2)	
C5	0.52935 (7)	0.16436 (11)	0.47481 (12)	0.02130 (19)	
H5A	0.5191	0.2088	0.5522	0.026*	
C6	0.46098 (6)	0.15033 (10)	0.34992 (11)	0.01791 (18)	
C7	0.37483 (7)	0.20586 (11)	0.34673 (11)	0.01807 (18)	
H7A	0.3655	0.2405	0.4294	0.022*	
C8	0.13003 (6)	0.06529 (10)	0.09358 (10)	0.01422 (16)	
C9	0.16949 (7)	−0.01247 (11)	0.00975 (11)	0.01837 (18)	
H9A	0.2011	0.0290	−0.0460	0.022*	
C10	0.16103 (7)	−0.15310 (11)	0.01051 (11)	0.01982 (19)	
H10A	0.1873	−0.2056	−0.0453	0.024*	
C11	0.11378 (6)	−0.21695 (10)	0.09355 (10)	0.01714 (17)	
C12	0.07307 (7)	−0.13615 (11)	0.17433 (11)	0.01804 (18)	
H12A	0.0400	−0.1773	0.2280	0.022*	
C13	0.08123 (6)	0.00428 (10)	0.17580 (10)	0.01632 (17)	
H13A	0.0545	0.0571	0.2308	0.020*	
C14	0.10817 (8)	−0.36948 (11)	0.09768 (13)	0.0235 (2)	
H14A	0.1204	−0.4070	0.0150	0.035*	
H14B	0.0499	−0.3958	0.1001	0.035*	
H14C	0.1506	−0.4030	0.1805	0.035*	
H1N2	0.2190 (11)	0.2514 (16)	0.3232 (17)	0.028 (4)*	
H1O1	0.3722 (14)	0.093 (2)	0.120 (2)	0.050 (6)*	

### Atomic displacement parameters (Å^2^)

**Table:**

	*U*^11^	*U*^22^	*U*^33^	*U*^12^	*U*^13^	*U*^23^
Cl1	0.01936 (12)	0.03239 (16)	0.03641 (16)	−0.00588 (10)	−0.00283 (10)	0.01246 (12)
S1	0.01639 (11)	0.01291 (11)	0.01269 (10)	0.00087 (7)	0.00572 (8)	0.00034 (7)
O1	0.0244 (4)	0.0326 (5)	0.0194 (4)	0.0074 (3)	0.0076 (3)	0.0000 (3)
O2	0.0311 (4)	0.0184 (3)	0.0158 (3)	0.0000 (3)	0.0114 (3)	0.0026 (3)
O3	0.0183 (3)	0.0169 (3)	0.0220 (3)	0.0041 (3)	0.0076 (3)	−0.0002 (3)
N1	0.0157 (3)	0.0144 (4)	0.0188 (4)	0.0000 (3)	0.0072 (3)	−0.0004 (3)
N2	0.0154 (3)	0.0173 (4)	0.0151 (3)	−0.0003 (3)	0.0064 (3)	−0.0029 (3)
C1	0.0208 (4)	0.0195 (5)	0.0215 (4)	0.0030 (4)	0.0079 (4)	0.0043 (4)
C2	0.0249 (5)	0.0272 (6)	0.0307 (5)	0.0076 (4)	0.0123 (4)	0.0047 (4)
C3	0.0197 (4)	0.0264 (6)	0.0384 (6)	0.0053 (4)	0.0101 (4)	0.0096 (5)
C4	0.0170 (4)	0.0210 (5)	0.0300 (5)	−0.0019 (4)	0.0026 (4)	0.0084 (4)
C5	0.0191 (4)	0.0194 (5)	0.0244 (5)	−0.0022 (4)	0.0042 (4)	0.0019 (4)
C6	0.0170 (4)	0.0156 (4)	0.0214 (4)	−0.0002 (3)	0.0059 (3)	0.0021 (3)
C7	0.0181 (4)	0.0169 (4)	0.0195 (4)	−0.0008 (3)	0.0057 (3)	−0.0020 (3)
C8	0.0148 (4)	0.0136 (4)	0.0140 (4)	0.0000 (3)	0.0036 (3)	−0.0005 (3)
C9	0.0231 (4)	0.0164 (4)	0.0182 (4)	−0.0009 (3)	0.0099 (3)	−0.0018 (3)
C10	0.0238 (4)	0.0165 (4)	0.0207 (4)	0.0007 (4)	0.0087 (4)	−0.0033 (3)
C11	0.0168 (4)	0.0145 (4)	0.0179 (4)	−0.0004 (3)	0.0009 (3)	0.0000 (3)
C12	0.0169 (4)	0.0177 (4)	0.0201 (4)	−0.0017 (3)	0.0060 (3)	0.0019 (3)
C13	0.0158 (4)	0.0168 (4)	0.0176 (4)	0.0002 (3)	0.0067 (3)	−0.0004 (3)
C14	0.0267 (5)	0.0151 (4)	0.0265 (5)	−0.0005 (4)	0.0037 (4)	0.0006 (4)

### Geometric parameters (Å, °)

**Table:**

Cl1—C4	1.7429 (12)	C5—H5A	0.9300
S1—O3	1.4296 (7)	C6—C7	1.4545 (14)
S1—O2	1.4386 (7)	C7—H7A	0.9300
S1—N2	1.6548 (9)	C8—C9	1.3905 (13)
S1—C8	1.7512 (10)	C8—C13	1.3954 (13)
O1—C1	1.3585 (14)	C9—C10	1.3895 (15)
O1—H1O1	0.75 (2)	C9—H9A	0.9300
N1—C7	1.2858 (13)	C10—C11	1.3965 (15)
N1—N2	1.3994 (11)	C10—H10A	0.9300
N2—H1N2	0.881 (17)	C11—C12	1.3982 (15)
C1—C2	1.3954 (15)	C11—C14	1.5037 (15)
C1—C6	1.4071 (15)	C12—C13	1.3866 (14)
C2—C3	1.3875 (18)	C12—H12A	0.9300
C2—H2A	0.9300	C13—H13A	0.9300
C3—C4	1.3886 (18)	C14—H14A	0.9600
C3—H3A	0.9300	C14—H14B	0.9600
C4—C5	1.3838 (15)	C14—H14C	0.9600
C5—C6	1.4010 (14)		
			
O3—S1—O2	120.16 (5)	C1—C6—C7	122.79 (9)
O3—S1—N2	103.86 (4)	N1—C7—C6	121.51 (9)
O2—S1—N2	106.09 (5)	N1—C7—H7A	119.2
O3—S1—C8	110.03 (5)	C6—C7—H7A	119.2
O2—S1—C8	109.02 (5)	C9—C8—C13	120.99 (9)
N2—S1—C8	106.73 (4)	C9—C8—S1	120.16 (7)
C1—O1—H1O1	107.2 (16)	C13—C8—S1	118.73 (7)
C7—N1—N2	115.26 (8)	C10—C9—C8	119.01 (9)
N1—N2—S1	114.07 (6)	C10—C9—H9A	120.5
N1—N2—H1N2	115.8 (11)	C8—C9—H9A	120.5
S1—N2—H1N2	110.5 (11)	C9—C10—C11	121.19 (9)
O1—C1—C2	117.97 (10)	C9—C10—H10A	119.4
O1—C1—C6	122.12 (9)	C11—C10—H10A	119.4
C2—C1—C6	119.91 (10)	C10—C11—C12	118.60 (9)
C3—C2—C1	120.33 (11)	C10—C11—C14	120.60 (10)
C3—C2—H2A	119.8	C12—C11—C14	120.79 (10)
C1—C2—H2A	119.8	C13—C12—C11	121.08 (9)
C2—C3—C4	119.61 (10)	C13—C12—H12A	119.5
C2—C3—H3A	120.2	C11—C12—H12A	119.5
C4—C3—H3A	120.2	C12—C13—C8	119.10 (9)
C5—C4—C3	121.01 (11)	C12—C13—H13A	120.4
C5—C4—Cl1	118.60 (10)	C8—C13—H13A	120.4
C3—C4—Cl1	120.38 (9)	C11—C14—H14A	109.5
C4—C5—C6	119.90 (11)	C11—C14—H14B	109.5
C4—C5—H5A	120.0	H14A—C14—H14B	109.5
C6—C5—H5A	120.0	C11—C14—H14C	109.5
C5—C6—C1	119.23 (9)	H14A—C14—H14C	109.5
C5—C6—C7	117.98 (9)	H14B—C14—H14C	109.5
			
C7—N1—N2—S1	−167.94 (8)	C5—C6—C7—N1	173.45 (10)
O3—S1—N2—N1	177.60 (7)	C1—C6—C7—N1	−7.19 (16)
O2—S1—N2—N1	−54.83 (8)	O3—S1—C8—C9	155.84 (8)
C8—S1—N2—N1	61.33 (8)	O2—S1—C8—C9	22.10 (10)
O1—C1—C2—C3	−179.18 (11)	N2—S1—C8—C9	−92.09 (8)
C6—C1—C2—C3	0.77 (17)	O3—S1—C8—C13	−28.14 (9)
C1—C2—C3—C4	−0.41 (18)	O2—S1—C8—C13	−161.88 (8)
C2—C3—C4—C5	−0.12 (18)	N2—S1—C8—C13	83.93 (8)
C2—C3—C4—Cl1	178.67 (9)	C13—C8—C9—C10	−1.10 (15)
C3—C4—C5—C6	0.28 (17)	S1—C8—C9—C10	174.83 (8)
Cl1—C4—C5—C6	−178.53 (8)	C8—C9—C10—C11	0.05 (16)
C4—C5—C6—C1	0.08 (16)	C9—C10—C11—C12	1.41 (16)
C4—C5—C6—C7	179.47 (10)	C9—C10—C11—C14	−177.60 (10)
O1—C1—C6—C5	179.34 (10)	C10—C11—C12—C13	−1.86 (15)
C2—C1—C6—C5	−0.60 (16)	C14—C11—C12—C13	177.14 (10)
O1—C1—C6—C7	−0.01 (16)	C11—C12—C13—C8	0.84 (15)
C2—C1—C6—C7	−179.95 (10)	C9—C8—C13—C12	0.66 (15)
N2—N1—C7—C6	−178.29 (9)	S1—C8—C13—C12	−175.32 (8)

### Hydrogen-bond geometry (Å, °)

**Table:**

*D*—H···*A*	*D*—H	H···*A*	*D*···*A*	*D*—H···*A*
O1—H1O1···N1	0.75 (2)	2.00 (2)	2.6690 (13)	149 (2)
N2—H1N2···O2^i^	0.881 (16)	1.961 (17)	2.8375 (12)	172.8 (17)
C7—H7A···O1^i^	0.93	2.59	3.3679 (14)	142
C10—H10A···Cl1^ii^	0.93	2.82	3.7256 (12)	164
C12—H12A···O3^iii^	0.93	2.48	3.3549 (14)	157

Symmetry codes: (i) *x*, −*y*+1/2, *z*+1/2; (ii) −*x*+1, *y*−1/2, −*z*+1/2; (iii) −*x*, *y*−1/2, −*z*+1/2.

## References

[bb1] Ali, H. M., Laila, M., Wan Jefrey, B. & Ng, S. W. (2007). *Acta Cryst.* E**63**, o1617–o1618.

[bb2] Allen, F. H., Kennard, O., Watson, D. G., Brammer, L., Orpen, A. G. & Taylor, R. (1987). *J. Chem. Soc. Perkin Trans. 2*, pp. S1–19.

[bb3] Bernstein, J., Davis, R. E., Shimoni, L. & Chang, N.-L. (1995). *Angew. Chem. Int. Ed. Engl.* **34**, 1555–1573.

[bb4] Bruker (2005). *APEX2*, *SAINT* and *SADABS* Bruker AXS Inc., Madison, Wisconsin, USA.

[bb5] Kayser, F. H., Bienz, K. A., Eckert, J. & Zinkernagel, R. M. (2004). *Medical Microbiology*, pp. 1–20. Berlin: Thieme Medical.

[bb6] Kia, R., Fun, H.-K. & Kargar, H. (2008). *Acta Cryst.* E**64**, o2341. 10.1107/S1600536808037069 PMC2959853 21581316

[bb7] Mehrabi, H., Kia, R., Hassanzadeh, A., Ghobadi, S. & Khavasi, H. R. (2008). *Acta Cryst.* E**64**, o1845. 10.1107/S1600536808027219 PMC2960482 21201816

[bb8] Sheldrick, G. M. (2008). *Acta Cryst.* A**64**, 112–122. 10.1107/S0108767307043930 18156677

[bb9] Spek, A. L. (2003). *J. Appl. Cryst.* **36**, 7–13.

[bb10] Tabatabaee, M., Anari-Abbasnejad, M., Nozari, N., Sadegheian, S. & Ghasemzadeh, M. (2007). *Acta Cryst.* E**63**, o2099–o2100.

[bb11] Tierney, L. M. Jr, McPhee, S. J. & Papadakis, M. A. (2006). *Current Medical Diagnosis & Treatment*, 45th ed., pp. 1–50. New York: McGraw-Hill Medical.

